# Supplementary material for: VDX-111 targets proliferative pathways in canine cancer cell lines
Source: PLoS One. 2024 May 21;19(5):e0303470. doi: 10.1371/journal.pone.0303470 (PMC11108205; doi:10.1371/journal.pone.0303470)
Supplement: S1 Raw images — (PDF) [file pone.0303470.s001.pdf]

S1 raw images
Figure 6 blots

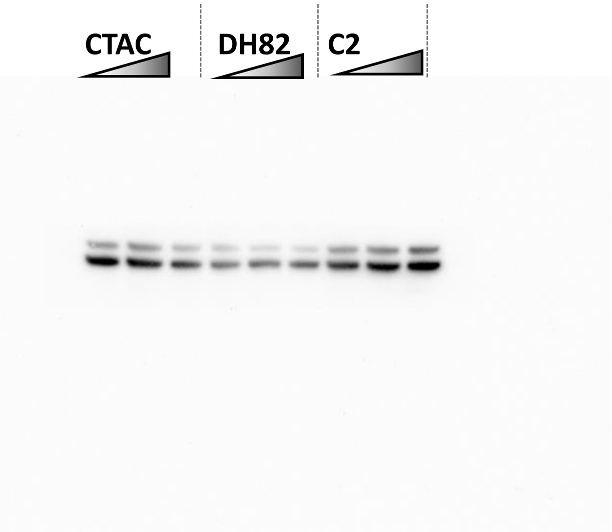

ERK1/2

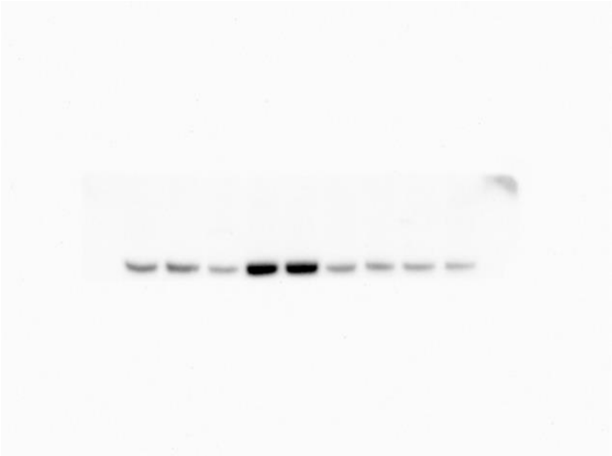

P-AKT

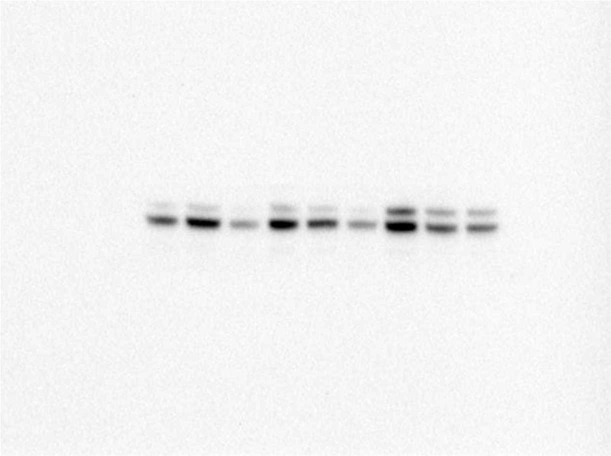

P-ERK1/2

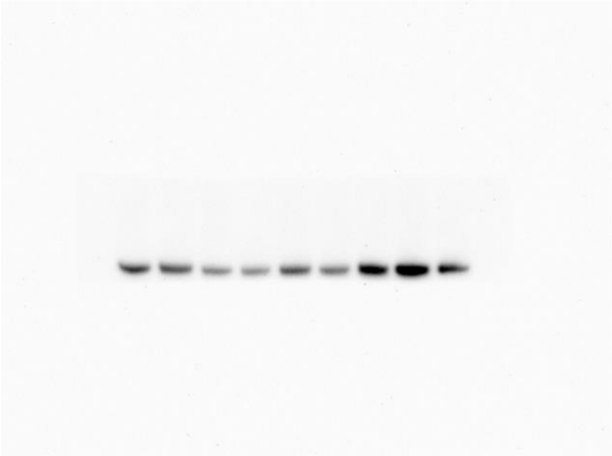

AKT

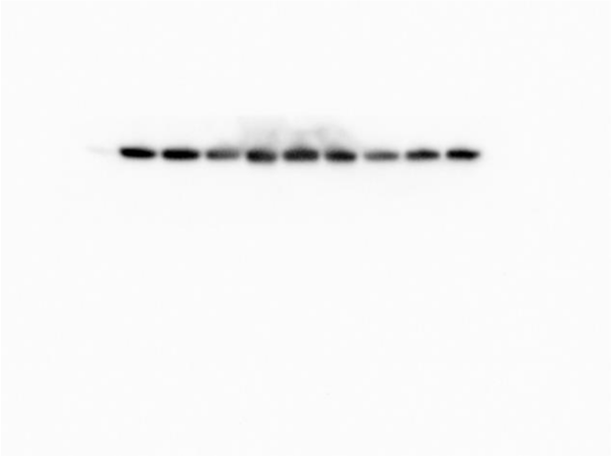

Cofilin

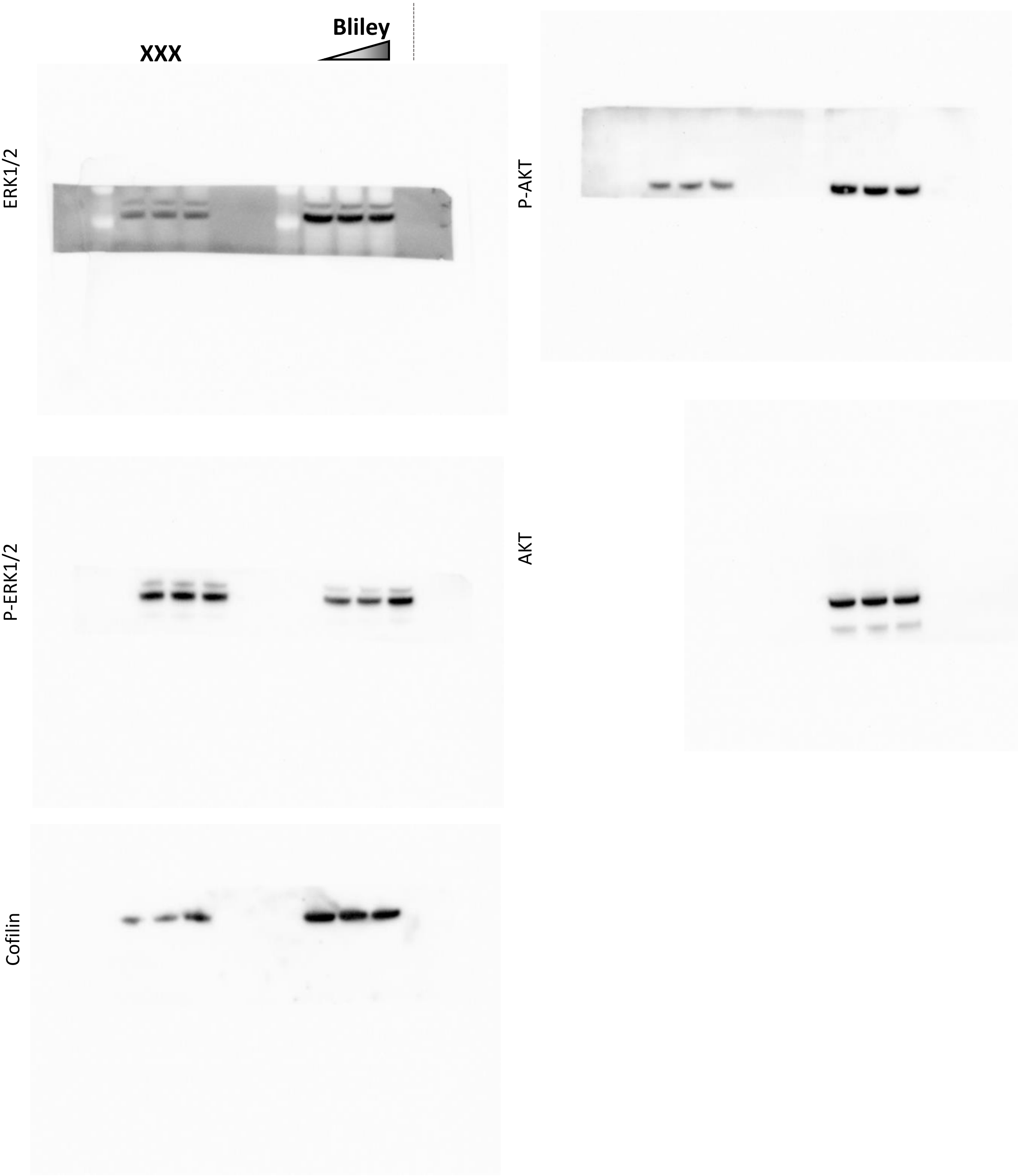
Bliley
XXX
ERK1/2
P-AKT
AKT
P-ERK1/2
Cofilin

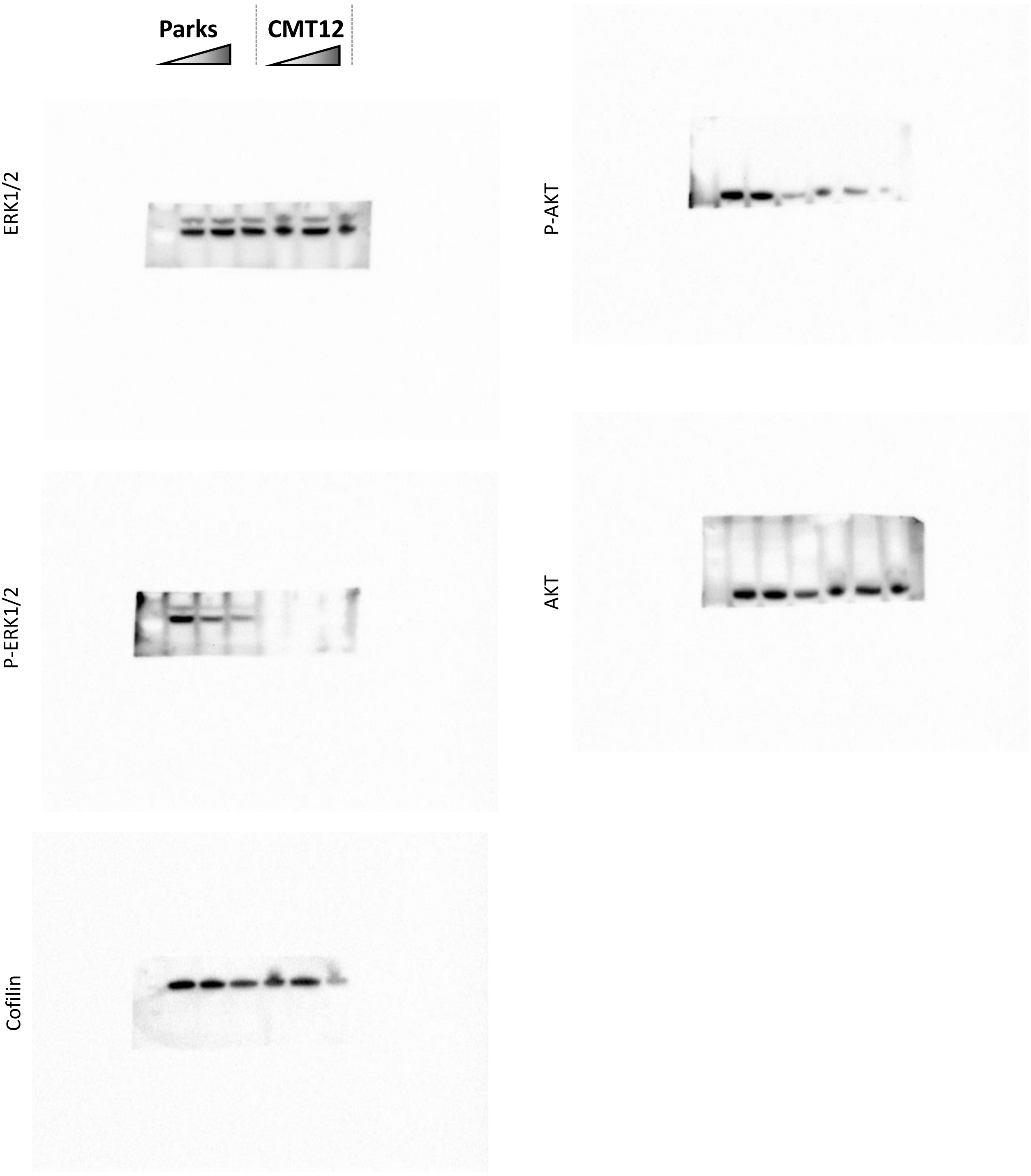
Parks
CMT12
ERK1/2
P-ERK1/2
Cofilin
P-AKT
AKT
