# Supplementary material for: VDX-111 targets proliferative pathways in canine cancer cell lines
Source: PLoS One. 2024 May 21;19(5):e0303470. doi: 10.1371/journal.pone.0303470 (PMC11108205; doi:10.1371/journal.pone.0303470)
Supplement: S1 File — A-C) Cells were exposed to VDX-111 or EtOH control for 72 hours before measuring PI fluorescence using flow cytometry. S2 Fig: VDX-111 sensitivity is not correlated with phosphatase expression despite established mechanism of action. A) Plots showing the FACC canine cell line panel PTPN3 expression correlating to survival at 1 μM and 100 nM. B) Plots showing the cell line panel PTP4A3 expression correlating to survival at 1 μM and 100 nM. S3 Fig: g:Profiler pathway analysis of gene expression correlating with VDX-111 sensitivity. Significantly correlated pathways for sensitivity at 1 μM. No pathways were significant for 100 nM. S4 Fig: Metascape pathway analysis of gene expression correlating with VDX-111 sensitivity. Significantly correlated pathways for sensitivity at 1 μM and 100 nM, inputting both positively and negatively correlated genes with a p>0.05. S5 Fig: Heatmap of expression levels in each cell line of genes in the “MAPK pathway”. Expression levels are displayed of the genes identified in the Metascape analysis of the MAPK pathway. Cell lines with >50% survival at 1 μM treatment are indicated as resistant, <50% survival at 1 μM treatment are indicated as sensitive. S6 Fig: GSEA of the top sensitive and most resistant cell lines. GSEA using normalized expression of 12,383 genes where I have compared untreated cell lines that were sensitive (CTAC, Cindy, SB) and resistant (CLL1390, DEN-HSA, Bliley, C2, BrMCT) to VDX-111. (PDF) [file pone.0303470.s002.pdf]

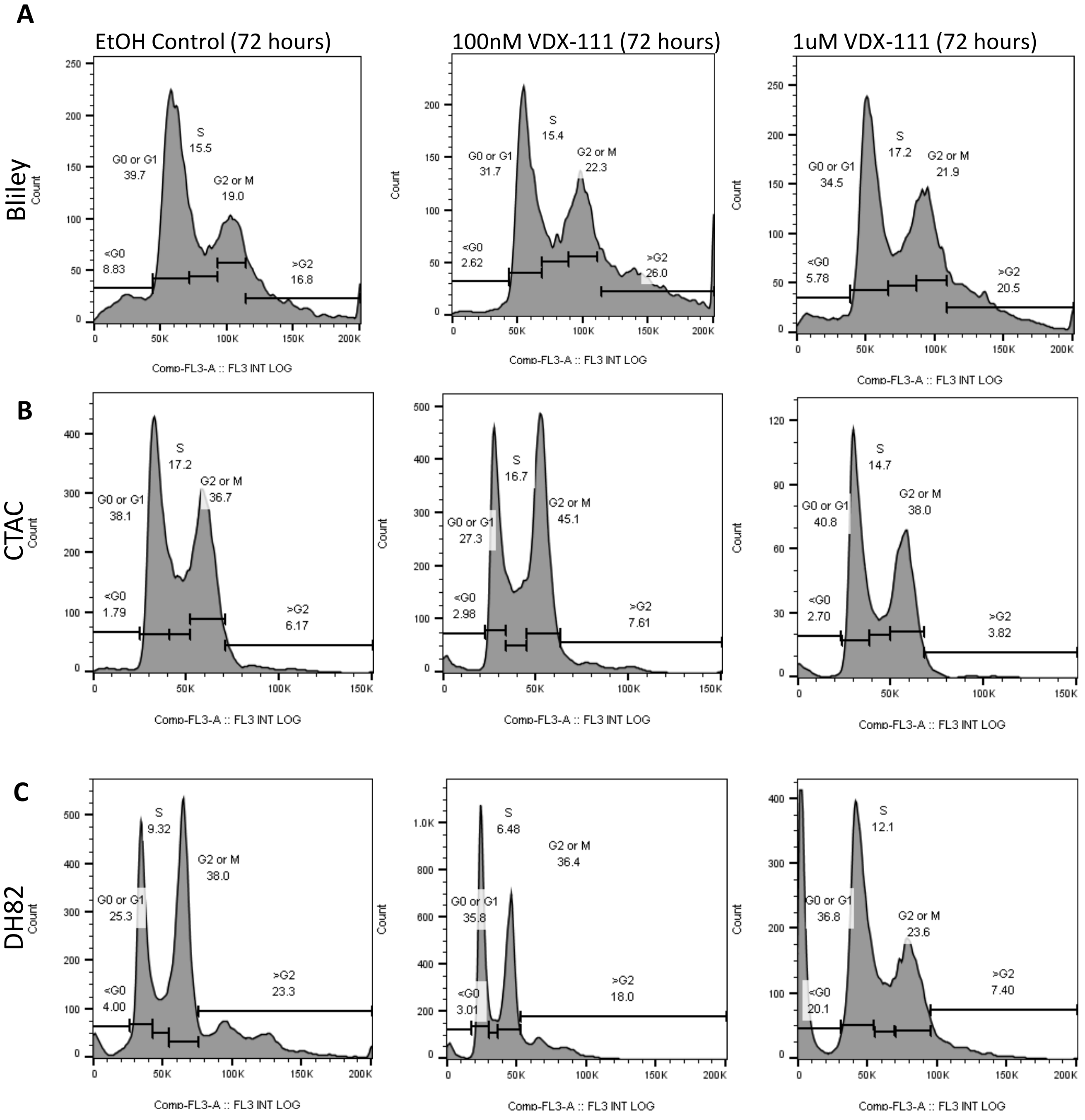
A
EtOH Control (72 hours)
100nM VDX-111 (72 hours)
1uM VDX-111 (72 hours)
Bliley
G0 or G1 39.7
S 15.5
G2 or M 19.0
<G0 8.83
>G2 16.8
G0 or G1 31.7
S 15.4
G2 or M 22.3
<G0 2.62
>G2 26.0
G0 or G1 34.5
S 17.2
G2 or M 21.9
<G0 5.78
>G2 20.5
B
CTAC
G0 or G1 38.1
S 17.2
G2 or M 36.7
<G0 1.79
>G2 6.17
G0 or G1 27.3
S 16.7
G2 or M 45.1
<G0 2.98
>G2 7.61
G0 or G1 40.8
S 14.7
G2 or M 38.0
<G0 2.70
>G2 3.82
C
DH82
G0 or G1 25.3
S 9.32
G2 or M 38.0
<G0 4.00
>G2 23.3
G0 or G1 35.8
S 6.48
G2 or M 36.4
<G0 3.01
>G2 18.0
G0 or G1 36.8
S 12.1
G2 or M 23.6
<G0 20.1
>G2 7.40
Count
Comp-FL3-A :: FL3 INT LOG

Figure S1

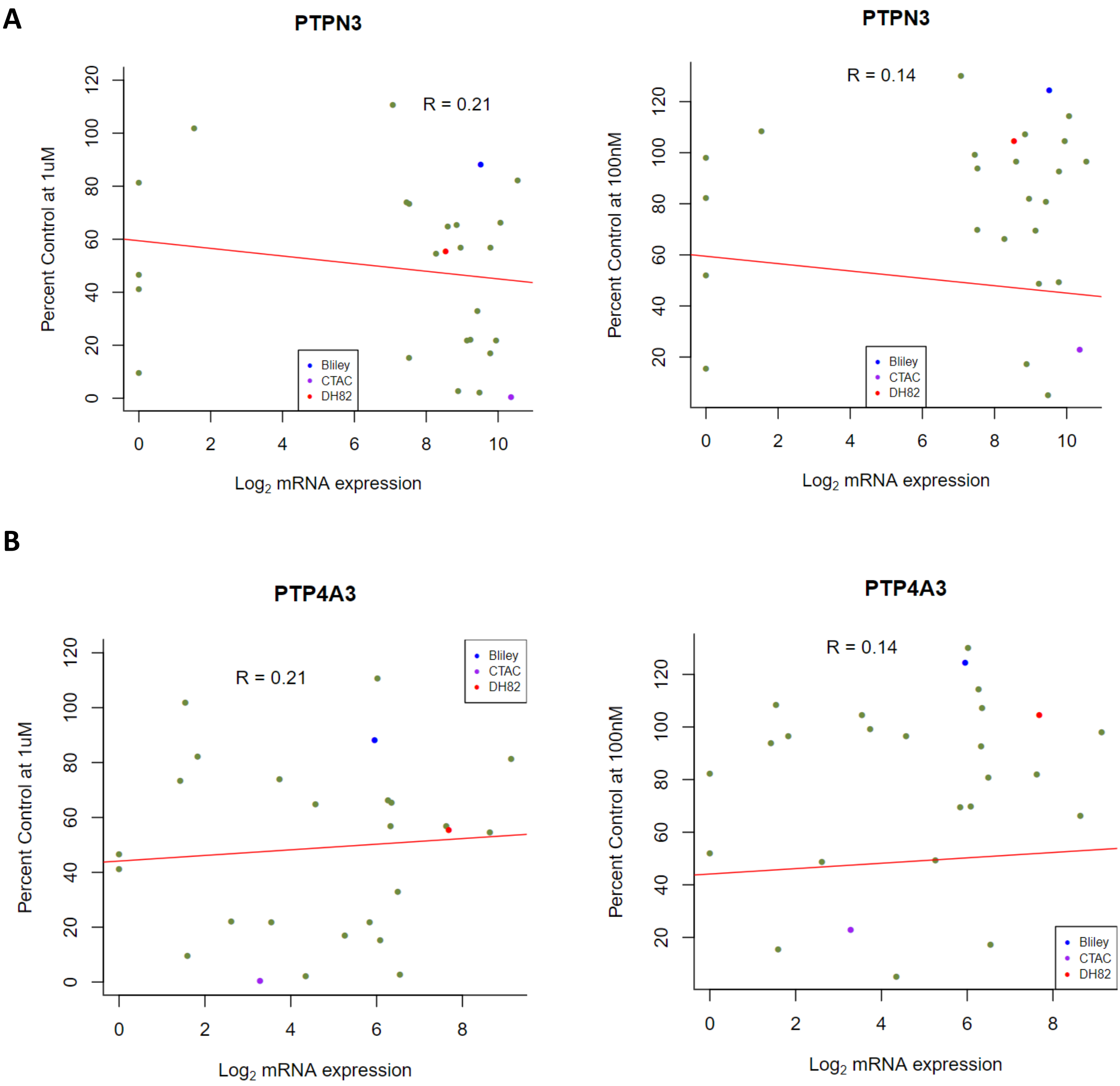

A
PTPN3
R = 0.21
Percent Control at 1uM
Log2 mRNA expression
Bliley
CTAC
DH82
PTPN3
R = 0.14
Percent Control at 100nM
Log2 mRNA expression
Bliley
CTAC
DH82
B
PTP4A3
R = 0.21
Percent Control at 1uM
Log2 mRNA expression
Bliley
CTAC
DH82
PTP4A3
R = 0.14
Percent Control at 100nM
Log2 mRNA expression
Bliley
CTAC
DH82

Figure S2

Figure S3

p-value	Pathway	
0.018515	RNA polymerase II transcription repressor complex	Positively associated genes
0.047659	chromatin	Positively associated genes
0.01837	Protein localization	Negatively associated genes
0.018697	Arginine and proline metabolism	Negatively associated genes
0.018697	Fat digestion and absorption	Negatively associated genes
0.042687	copper ion binding	Negatively associated genes

1 µM VDX-111

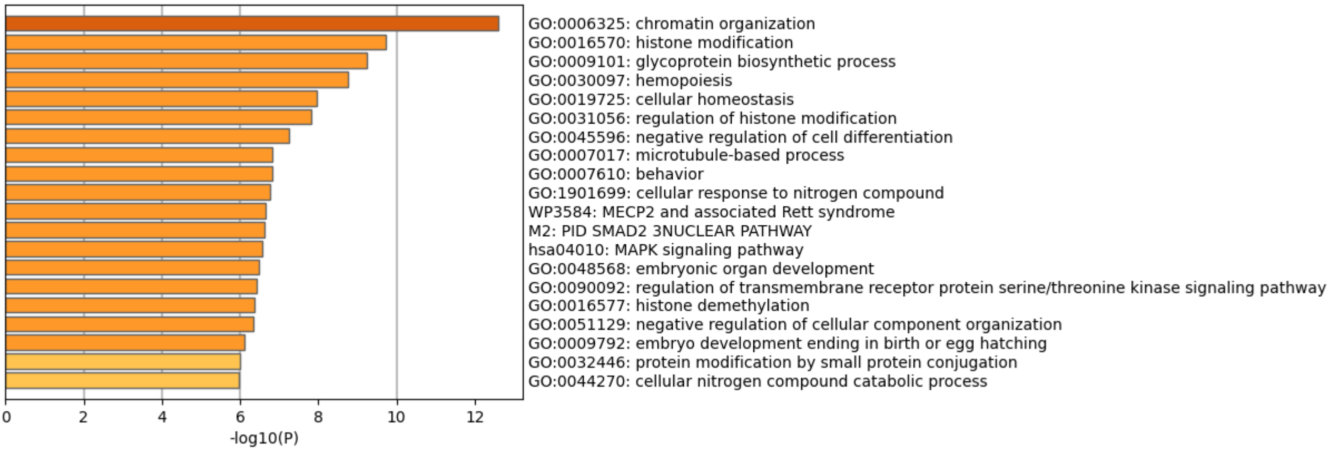

100 nM VDX-111

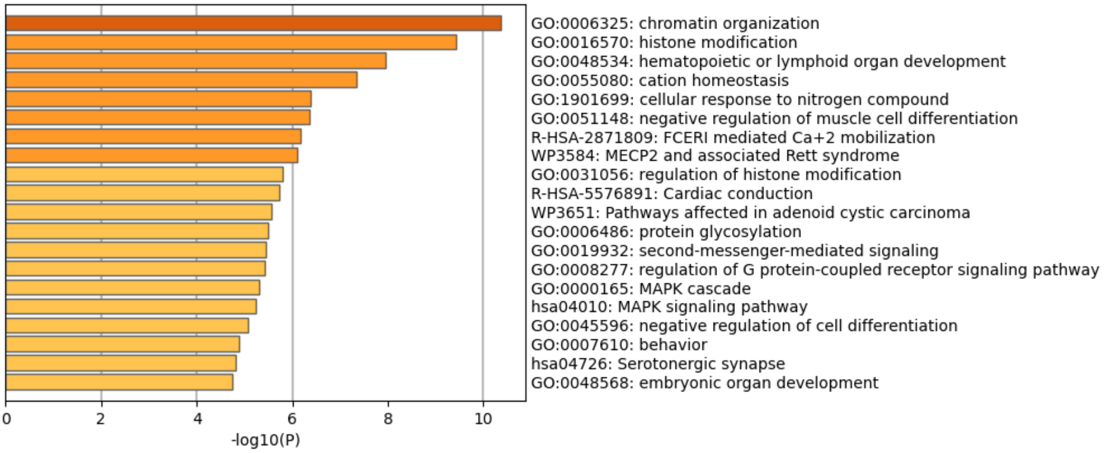

Figure S4

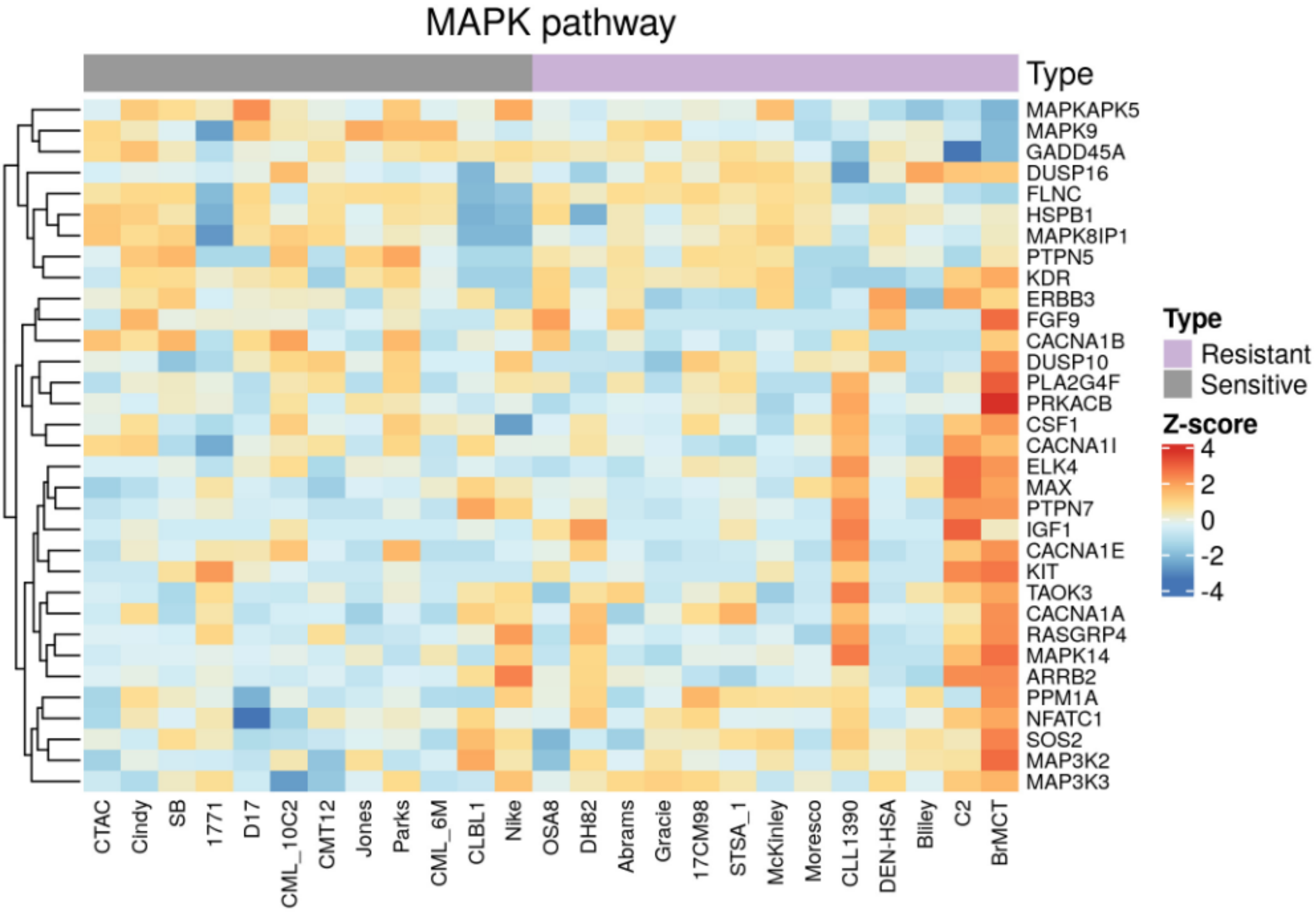
MAPK pathway
Type
MAPKAPK5
MAPK9
GADD45A
DUSP16
FLNC
HSPB1
MAPK8IP1
PTPN5
KDR
ERBB3
FGF9
CACNA1B
DUSP10
PLA2G4F
PRKACB
CSF1
CACNA1I
ELK4
MAX
PTPN7
IGF1
CACNA1E
KIT
TAOK3
CACNA1A
RASGRP4
MAPK14
ARRB2
PPM1A
NFATC1
SOS2
MAP3K2
MAP3K3
CTAC
Cindy
SB
1771
D17
CML_10C2
CMT12
Jones
Parks
CML_6M
CLBL1
Nike
OSA8
DH82
Abrams
Gracie
17CM98
STSA_1
McKinley
Moresco
CLL1390
DEN-HSA
Bliley
C2
BrMCT
Type
Resistant
Sensitive
Z-score
4
2
0
-2
-4

Figure S5

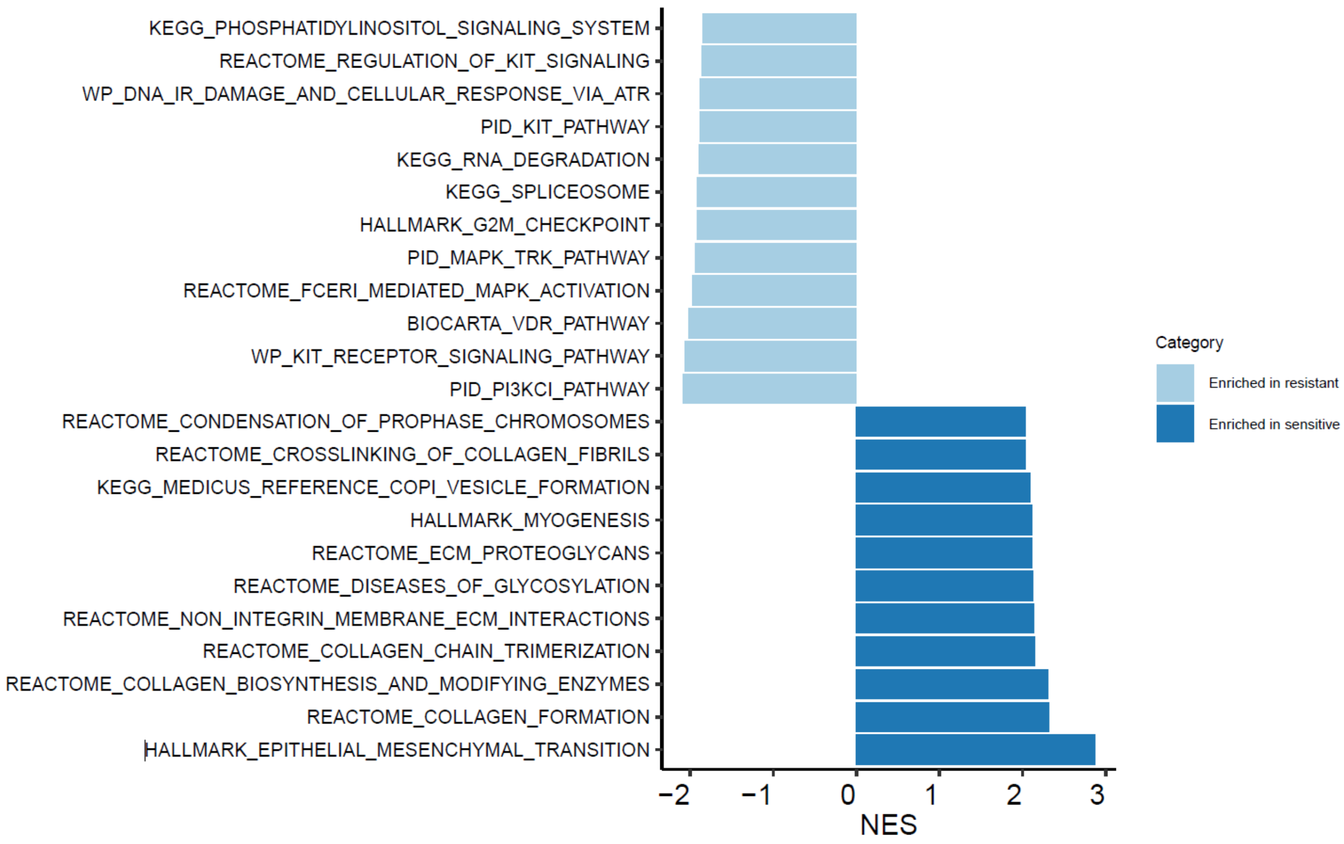

KEGG_PHOSPHATIDYLINOSITOL_SIGNALING_SYSTEM
REACTOME_REGULATION_OF_KIT_SIGNALING
WP_DNA_IR_DAMAGE_AND_CELLULAR_RESPONSE_VIA_ATR
PID_KIT_PATHWAY
KEGG_RNA_DEGRADATION
KEGG_SPLICEOSOME
HALLMARK_G2M_CHECKPOINT
PID_MAPK_TRK_PATHWAY
REACTOME_FCERI_MEDIATED_MAPK_ACTIVATION
BIOCARTA_VDR_PATHWAY
WP_KIT_RECEPTOR_SIGNALING_PATHWAY
PID_PI3KCI_PATHWAY
REACTOME_CONDENSATION_OF_PROPHASE_CHROMOSOMES
REACTOME_CROSSLINKING_OF_COLLAGEN_FIBRILS
KEGG_MEDICUS_REFERENCE_COPI_VESICLE_FORMATION
HALLMARK_MYOGENESIS
REACTOME_ECM_PROTEOGLYCANS
REACTOME_DISEASES_OF_GLYCOSYLATION
REACTOME_NON_INTEGRIN_MEMBRANE_ECM_INTERACTIONS
REACTOME_COLLAGEN_CHAIN_TRIMERIZATION
REACTOME_COLLAGEN_BIOSYNTHESIS_AND_MODIFYING_ENZYMES
REACTOME_COLLAGEN_FORMATION
HALLMARK_EPITHELIAL_MESENCHYMAL_TRANSITION
−2
−1
0
1
2
3
NES
Category
Enriched in resistant
Enriched in sensitive

Figure S6

SUPPLEMENTAL FIGURE LEGENDS:

S1 Fig: VDX-111 has minimal affect on cell cycle distribution. A-C) Cells were exposed to VDX-111 or EtOH control for 72 hours before measuring PI fluorescence using flow cytometry.

S2 Fig: VDX-111 sensitivity is not correlated with phosphatase expression despite established mechanism of action. A) Plots showing the FACC canine cell line panel PTPN3 expression correlating to survival at 1 µM and 100 nM. B) Plots showing the cell line panel PTP4A3 expression correlating to survival at 1 µM and 100 nM.

S3 Fig: g:Profiler pathway analysis of gene expression correlating with VDX-111 sensitivity. Significantly correlated pathways for sensitivity at 1 µM. No pathways were significant for 100 nM.

S4 Fig: Metascape pathway analysis of gene expression correlating with VDX-111 sensitivity. Significantly correlated pathways for sensitivity at 1 µM and 100 nM, inputting both positively and negatively correlated genes with a $p>0.05$.

S5 Fig: Heatmap of expression levels in each cell line of genes in the "MAPK pathway". Expression levels are displayed of the genes identified in the Metascape analysis of the MAPK pathway. Cell lines with >50% survival at 1 µM treatment are indicated as resistant, <50% survival at 1 µM treatment are indicated as sensitive.

S6 Fig: GSEA of the top sensitive and most resistant cell lines. GSEA using normalized expression of 12,383 genes where I have compared untreated cell lines that were sensitive (CTAC, Cindy, SB) and resistant (CLL1390, DEN-HSA, Bliley, C2, BrMCT) to VDX-111.

Supplemental videos: Videos of experiments quantified in Figure 2; (S1 Video) Bliley (NucRed) + 100 nM VDX-111 and 100 nM YOYO1, (S2 Video) CTAC (NucRed) + 100 nM VDX-111 and 100 nM YOYO1, (S3 Video) DH82 (NucRed) + 100 nM VDX-111 and 100 nM YOYO1

S1 Table: **Canine genes with expression correlated to VDX-111 survival**.

S2 Table: Pairwise comparison of cell lines. Sensitive (CTAC, Cindy, SB) and resistant (CLL1390, DEN-HSA, Bliley, C2, BrMCT) cell lines were compared using DESeq2, and differentially expressed genes with adjusted p-value or FDR 1.5 & <(-1.5).

S3 Table: GSEA enriched gene sets in sensitive and resistant cell lines.
